# Discharge Cognitive–Motor Imbalance Patterns and Long-Term Outcomes After Traumatic Brain Injury: A Propensity Score-Matched Cohort Study

**DOI:** 10.3390/jcm15093249

**Published:** 2026-04-24

**Authors:** Ji Hyun Kim, Seo Young Kim, Tae Uk Kim, Jung Keun Hyun, Sung Ryul Shim, Yuna Kim

**Affiliations:** 1 Department of Rehabilitation Medicine, College of Medicine, Dankook University, Cheonan 31116, Republic of Korea; jh0607@dkuh.co.kr (J.H.K.); juliet8383@naver.com (S.Y.K.); magnarbor@dankook.ac.kr (T.U.K.); 2 Department of Physical Medicine & Rehabilitation, School of Medicine, Ajou University, Suwon 16499, Republic of Korea; rhhyun@ajou.ac.kr; 3 Department of Biomedical Science, School of Medicine, Ajou University, Suwon 16499, Republic of Korea; 4 Department of Biomedical Informatics, College of Medicine, Konyang University, Daejeon 35365, Republic of Korea

**Keywords:** traumatic brain injury, rehabilitation, disability rating scale, functional independence measure, participation

## Abstract

**Background/Objectives**: Traumatic brain injury (TBI) often results in heterogeneous recovery across cognitive and motor domains. However, the prognostic implications of motor–cognitive imbalance at the time of rehabilitation discharge remain unclear. This study investigated whether discharge patterns of motor–cognitive imbalance are associated with functional outcomes at 1- and 2-year follow-up in individuals with moderate-to-severe TBI. **Methods**: We conducted a retrospective cohort study using the Traumatic Brain Injury Model Systems (TBIMS) National Database. Adults discharged from inpatient rehabilitation with available Functional Independence Measure (FIM) motor and cognitive subscores were included (*n* = 8342). Participants were classified as cognitive-dominant, motor-dominant, or balanced based on standardized discrepancies between FIM motor and cognitive scores. Propensity score matching was performed against the balanced group, yielding a matched cohort of 1310 participants. Outcomes included the Disability Rating Scale (DRS), FIM (total and subscales), Glasgow Outcome Scale—Extended (GOSE), and Participation Assessment with Recombined Tools—Objective (PART-O) at 1 and 2 years. **Results**: The matched cohort included cognitive-dominant (*n* = 524), motor-dominant (*n* = 524), and balanced (*n* = 262) participants with good covariate balance. Compared with the balanced group, the cognitive-dominant group showed lower disability severity (DRS) at 1 year (β = −0.45; 95% CI, −0.81 to −0.09) and 2 years (β = −0.40; 95% CI, −0.78 to −0.03), and higher FIM total scores at both time points. The motor-dominant group demonstrated higher FIM motor scores but lower odds of favorable disability status (DRS ≤ 3) at 2 years (OR = 0.52; 95% CI, 0.31–0.88). GOSE and PART-O outcomes did not differ significantly across groups. **Conclusions**: Discharge motor–cognitive imbalance patterns were associated with modest but distinct differences in long-term disability severity and functional independence after moderate-to-severe TBI. The direction of imbalance may provide additional prognostic context beyond total functional scores and support domain-targeted post-acute rehabilitation planning.

## 1. Introduction

Traumatic brain injury (TBI) is a major cause of death and disability worldwide and imposes a substantial long-term burden through chronic disability, reduced quality of life, and lost productivity [1,2,3,4]. In inpatient rehabilitation, clinicians routinely assess disability and functional independence at discharge using standardized instruments such as the Disability Rating Scale (DRS) [5] and the Functional Independence Measure (FIM) [6]. Longer-term outcomes are commonly characterized using the Glasgow Outcome Scale—Extended (GOSE) [7] and the Participation Assessment with Recombined Tools—Objective (PART-O) [8].

Recovery after moderate-to-severe TBI is highly heterogeneous. Although early injury severity indicators, such as the Glasgow Coma Scale (GCS), are established predictors of outcome, they do not fully explain interindividual variability in long-term disability, independence, and participation [9]. Moreover, even among patients with similar overall functional severity at rehabilitation discharge, subsequent recovery trajectories may diverge, suggesting that prognostically relevant information may not be fully captured by global severity indices alone.

Cognitive and motor impairments represent distinct determinants of long-term functioning after TBI [10,11,12,13]. Cognitive deficits in attention, memory, and executive function may limit independent living even when physical recovery is relatively favorable [10,11], whereas motor impairments may restrict mobility and self-care despite relatively preserved cognition [12,13]. However, commonly used composite functional measures may obscure within-person discrepancies between cognitive and motor domains [14,15]. As a result, individuals with similar total functional scores may have markedly different distributions of cognitive and motor impairment. This study extends prior research by explicitly examining within-person cognitive–motor imbalance at discharge, rather than relying solely on global functional severity indices, and evaluates whether this domain-specific discrepancy provides additional prognostic information for long-term outcomes.

Therefore, using the Traumatic Brain Injury Model Systems (TBIMS) National Database [16,17], this study aimed to (1) classify individuals with moderate-to-severe TBI according to discharge cognitive–motor patterns based on FIM cognitive and FIM motor subscores; (2) compare disability, functional independence, global outcome, and participation at 1 and 2 years post-injury across these patterns using propensity score-matched analyses; and (3) explore whether these associations differed according to discharge severity strata.

## 2. Materials and Methods

### 2.1. Study Design and Data Source

This retrospective cohort study used data from the TBIMS National Database, a prospective multicenter registry of individuals with moderate-to-severe TBI treated at participating inpatient rehabilitation centers in the United States [16,17].

The TBIMS program comprises a network of specialized rehabilitation centers across the United States, including 16 federally funded TBIMS Centers and 4 Longitudinal Follow-Up Centers, providing nationwide coverage of TBI care and outcomes research. Data collection is conducted using standardized protocols and operating procedures established by the TBIMS National Data and Statistical Center to ensure consistency across sites. Discharge measures are obtained as part of routine clinical documentation during inpatient rehabilitation, whereas follow-up outcomes are collected through structured interviews administered by trained personnel at each center.

The TBIMS program collects standardized data on demographics, injury characteristics, care processes, and outcomes from acute hospitalization through long-term follow-up [16,17]. We used the TBIMS public-use dataset released on 5 November 2025. This study was reported in accordance with the Strengthening the Reporting of Observational Studies in Epidemiology (STROBE) guidance for propensity score analysis [18].

### 2.2. Participants

We included adults aged ≥16 years in the TBIMS National Database without restriction on injury year. Eligibility required available FIM motor and FIM cognitive subscale scores at discharge from inpatient rehabilitation.

For outcome analyses, a complete-case approach was applied at each follow-up time point. Participants were included in each model if they had non-missing data for the outcome of interest at 1 or 2 years and complete baseline covariates required for propensity score estimation. Participants with missing baseline covariates were excluded.

### 2.3. Exposure: Discharge Cognitive–Motor Imbalance Pattern

The primary exposure was discharge cognitive–motor imbalance, defined using the FIM motor and FIM Cognitive subscales [6]. The FIM motor subscale (range, 13–91) evaluates self-care, sphincter control, transfers, and locomotion, whereas the FIM cognitive subscale (range, 5–35) evaluates communication and social cognition [6].

Each subscale was rescaled to a 0–100 scale. This approach expresses each subscale as a proportion of its achievable range, thereby accounting for differences in scale width rather than comparing raw score differences across subscales with different ranges. A standardized discrepancy score was then calculated as the rescaled FIM cognitive score minus the rescaled FIM motor score, divided by the sample standard deviation of the discrepancy. This rescaling was used only for defining the exposure and was not applied in outcome analyses, which were conducted using the original, unrescaled measures.

Using an a priori threshold of ±0.15 standard deviations (SDs), participants were classified as cognitive-dominant if the discrepancy favored cognition by ≥0.15 SD, motor-dominant if it favored motor function by ≤−0.15 SD, and balanced if the discrepancy was within ±0.15 SD. The ±0.15 SD threshold was selected to represent a small effect size according to Cohen’s convention [19] while preserving sample size for stable matching. These categories were treated as operational phenotypes based on an a priori definition rather than validated clinical cutoffs, and should be interpreted as providing contextual, research-oriented information rather than deterministic clinical classifications.

### 2.4. Outcome Measures

#### 2.4.1. Primary Outcome

The primary outcome was global disability measured by the DRS (range, 0–29), with higher scores indicating greater disability [5]. The DRS was analyzed as a continuous outcome. Favorable disability status was additionally defined a priori as DRS ≤ 3.

#### 2.4.2. Secondary Outcome

Functional independence at 1 and 2 years was assessed using the FIM total score (range, 18–126) and the FIM motor and FIM cognitive subscales [6]. Higher scores indicate greater independence.

#### 2.4.3. Exploratory Outcomes

Global functional status and societal participation were treated as exploratory outcomes because they are influenced by broader contextual factors.

Global functional outcome was assessed using the Glasgow Outcome Scale—Extended (GOSE), an 8-point ordinal scale ranging from death (1) to upper good recovery (8), with higher scores indicating better recovery [7,20]. For comparability with prior TBIMS studies, GOSE was analyzed as an approximately continuous outcome.

Societal participation was assessed using the Participation Assessment with Recombined Tools—Objective (PART-O), which includes three domains (Out and About, Productivity, and Social), each scored from 0 to 5, with higher scores indicating greater participation [8].

All follow-up outcomes were collected at 1 and 2 years post-injury using standardized structured interviews.

### 2.5. Covariates

Baseline covariates were selected a priori based on clinical relevance, prior literature, and data completeness [10,11,12,13,16,17]. These included demographic variables (age, sex, race/ethnicity, marital status, education level, and preinjury employment status) and injury-related variables (injury mechanism, admission GCS score, and history of problematic alcohol use). Discharge FIM total score was included to account for overall functional severity [6,16], such that the estimated associations reflected imbalance patterns beyond global discharge status.

### 2.6. Propensity Score Matching

To reduce confounding due to baseline differences across discharge patterns, propensity score matching was performed using the balanced group as the common reference. Propensity scores were estimated using two separate multivariable logistic regression models comparing (1) cognitive-dominant versus balanced and (2) motor-dominant versus balanced, each including all baseline covariates described above [21,22,23,24].

For each comparison, 1:2 nearest-neighbor matching without replacement was performed, such that one balanced participant was matched to two unbalanced participants, using a caliper width of 0.25 times the SD of the logit of the propensity score [23]. Covariate balance after matching was evaluated using standardized mean differences (SMDs), with |SMD| < 0.10 considered indicative of adequate balance [25,26].

The final analytic cohort was created by combining the two matched samples with the balanced group retained as the common comparator. Duplicate balanced participants were removed. Primary inferences focused on each imbalance group relative to the balanced reference group. Direct comparisons between the cognitive-dominant and motor-dominant groups were considered exploratory.

### 2.7. Statistical Analysis

Analyses were conducted using a complete-case approach at each time point, such that the analytic sample may vary across outcomes and time points and may differ from the originally matched cohort. In the matched cohort, continuous outcomes (DRS, FIM, GOSE, and PART-O) were analyzed using linear regression, and the binary outcome (DRS ≤ 3 vs. >3) was analyzed using logistic regression. Exposure was modeled as a three-level indicator variable with the balanced group as the reference category. To improve precision and address residual imbalance, all regression models were additionally adjusted for the same covariates included in the propensity score models, including discharge FIM total score.

Effect modification by discharge functional status was explored using stratified analyses. DRS strata were defined using established thresholds as severe (≥7), moderate (4–6), and mild (≤3). Discharge FIM total strata (<60, 60–90, and >90) were prespecified for interpretability in sensitivity analyses and were treated as exploratory rather than validated severity cutoffs. The same modeling strategy was applied within each stratum, and subgroup findings were interpreted cautiously. Statistical significance was defined as a two-sided *p* < 0.05.

All analyses were performed using R software (version 4.3.3; R Foundation for Statistical Computing, Vienna, Austria) with the MatchIt package. Figures were generated using Python (version 3.10) with matplotlib and seaborn.

## 3. Results

### 3.1. Participant Characteristics and Propensity Score Matching

Before propensity score matching, 8342 individuals met the discharge eligibility criteria, including 974 in the balanced group, 2484 in the cognitive-dominant group, and 4884 in the motor-dominant group. The motor-dominant pattern was the most common profile (59%). Compared with the balanced group, the cognitive-dominant group more frequently had motor vehicle crash as the injury mechanism and had slightly lower discharge FIM total scores. The motor-dominant group was younger, more often male, and had the highest discharge FIM total scores (Table 1).

Separate propensity score models were constructed for each pairwise comparison (cognitive-dominant versus balanced and motor-dominant versus balanced), followed by 1:2 matching and recombination of the matched sets. The final matched analytic cohort included 1310 participants: 262 balanced, 524 cognitive-dominant, and 524 motor-dominant. After matching, baseline covariates, including discharge FIM total score, were well balanced across groups (all SMDs < 0.10), and the propensity score distributions showed improved overlap between matched groups (Table 1 and Supplementary Figure S1).

### 3.2. Primary and Secondary Outcomes

**Figure 1:**
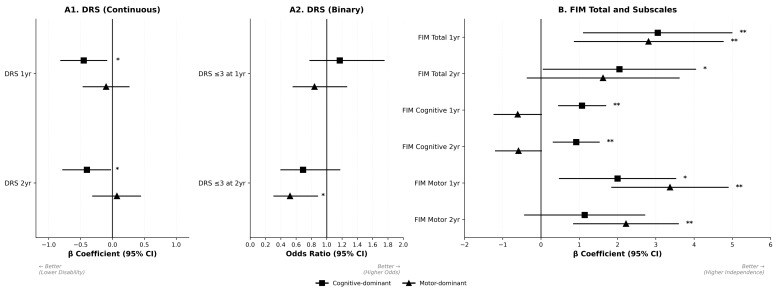
Associations of discharge cognitive–motor imbalance patterns with primary and secondary outcomes. Adjusted associations of cognitive-dominant and motor-dominant discharge imbalance patterns with 1-year and 2-year outcomes are shown relative to the balanced reference group. Panel (**A1**) shows continuous Disability Rating Scale (DRS) outcomes as linear regression coefficients (β) with 95% confidence intervals (CIs), where negative β indicates lower disability (better outcome). Panel (**A2**) shows binary DRS outcomes (DRS ≤ 3) as odds ratios (ORs) with 95% CIs, where OR > 1 indicates higher odds of a favorable outcome. Panel (**B**) shows Functional Independence Measure (FIM) total and subscale outcomes as β with 95% CIs, where positive β indicates greater functional independence. Squares represent cognitive-dominant estimates and triangles represent motor-dominant estimates. Horizontal bars indicate 95% CIs. * *p* < 0.05; ** *p* < 0.01. Abbreviations: CI, confidence interval; DRS, Disability Rating Scale; FIM, Functional Independence Measure.

Adjusted associations between discharge cognitive–motor imbalance patterns and 1- and 2-year outcomes are shown in Figure 1 and Table 2. Continuous outcomes are presented as regression coefficients (β), with lower values indicating better outcomes for DRS and higher values indicating better outcomes for FIM. Binary disability outcomes (DRS ≤ 3) are presented as odds ratios (ORs), with OR > 1 indicating higher odds of a favorable outcome. All models were adjusted for age, sex, race, marital status, education, employment, injury mechanism, GCS total score, alcohol history, and discharge FIM total score.

#### 3.2.1. Cognitive-Dominant Versus Balanced

Compared with the balanced group, the cognitive-dominant group had lower DRS scores at 1 year (β = −0.45; 95% confidence interval [CI], −0.81 to −0.09; *p* = 0.02) and 2 years (β = −0.40; 95% CI, −0.78 to −0.03; *p* = 0.03). FIM total scores were higher at 1 year (β = 3.05; 95% CI, 1.11 to 4.99; *p* = 0.002) and 2 years (β = 2.05; 95% CI, 0.06 to 4.04; *p* = 0.04). FIM cognitive scores were also higher at 1 year (β = 1.07; 95% CI, 0.46 to 1.69; *p* < 0.001) and 2 years (β = 0.92; 95% CI, 0.32 to 1.52; *p* = 0.002). FIM motor score was higher at 1 year (β = 2.00; 95% CI, 0.48 to 3.52; *p* = 0.010) but not at 2 years (β = 1.14; 95% CI, −0.43 to 2.71; *p* = 0.16). The odds of achieving DRS ≤ 3 did not differ significantly at 1 year (OR = 1.17; 95% CI, 0.78 to 1.75; *p* = 0.45) or 2 years (OR = 0.69; 95% CI, 0.40 to 1.17; *p* = 0.17).

#### 3.2.2. Motor-Dominant Versus Balanced

Compared with the balanced group, the motor-dominant group did not differ significantly in continuous DRS score at 1 year (β = −0.10; 95% CI, −0.46 to 0.26; *p* = 0.60) or 2 years (β = 0.07; 95% CI, −0.31 to 0.44; *p* = 0.72). FIM total score was higher at 1 year (β = 2.81; 95% CI, 0.87 to 4.76; *p* = 0.005) but not at 2 years (β = 1.62; 95% CI, −0.36 to 3.61; *p* = 0.11). FIM motor scores were higher at 1 year (β = 3.37; 95% CI, 1.85 to 4.89; *p* < 0.001) and 2 years (β = 2.22; 95% CI, 0.85 to 3.59; *p* = 0.002), whereas FIM cognitive scores were lower at both time points (1 year: β = −0.61; 95% CI, −1.23 to 0.01; *p* = 0.05; 2 years: β = −0.59; 95% CI, −1.19 to 0.01; *p* = 0.05). For binary disability outcomes, the motor-dominant group had lower odds of DRS ≤ 3 at 2 years (OR = 0.52; 95% CI, 0.31 to 0.88; *p* = 0.01), whereas the 1-year odds did not differ significantly (OR = 0.84; 95% CI, 0.56 to 1.26; *p* = 0.40).

### 3.3. Exploratory Outcomes

No significant differences were observed in GOSE or PART-O domain scores between either imbalance group and the balanced group at 1 or 2 years (Table 3).

### 3.4. Stratified Analyses by Discharge Severity

In stratified analyses, between-group differences were generally modest but were more apparent in moderate discharge severity strata, particularly among participants with discharge FIM total scores of 60–90 (Supplementary Table S1). In this stratum, the cognitive-dominant group had higher 1-year FIM total score (β = 2.38; 95% CI, 0.14 to 4.62; *p* = 0.04) and higher 1-year FIM cognitive score (β = 1.15; 95% CI, 0.33 to 1.96; *p* = 0.006) than the balanced group. In the same stratum, the motor-dominant group had higher 1-year FIM total score (β = 2.52; 95% CI, 0.27 to 4.77; *p* = 0.03) and higher 1-year FIM motor score (β = 3.16; 95% CI, 1.53 to 4.80; *p* < 0.001). Across DRS-based strata, effect estimates were generally directionally consistent with the overall results, although confidence intervals were wider in the severe strata.

## 4. Discussion

In this large multicenter cohort of adults with moderate-to-severe TBI who underwent specialized inpatient rehabilitation, discharge cognitive–motor imbalance patterns were associated with distinct but modest differences in long-term functional outcomes [15]. After propensity score matching and covariate adjustment, the cognitive-dominant pattern was associated with lower disability severity and greater functional independence at 1 and 2 years compared with the balanced pattern. In contrast, the motor-dominant pattern was associated with better motor independence but not with lower overall disability severity and was associated with lower odds of favorable disability status at 2 years. These findings suggest that, among individuals with comparable overall discharge severity, the direction of cognitive–motor imbalance may provide clinically relevant information beyond global severity indices alone.

The more favorable trajectory observed in the cognitive-dominant group suggests that relatively preserved cognitive capacity at discharge may support adaptation during long-term recovery [15]. Cognitive abilities may facilitate strategy learning, adherence to rehabilitation recommendations, and self-management after return to the community. Although the effect sizes were modest, the consistent pattern across DRS and FIM outcomes indicates that cognitive resources at discharge may modify functional trajectories even when overall severity is similar. This interpretation is consistent with the concept of cognitive reserve, whereby preserved cognitive resources may support the acquisition and generalization of compensatory strategies from structured rehabilitation settings to real-world environments [27,28].

The motor-dominant profile reflects a clinically important mismatch between visible physical recovery and less apparent cognitive vulnerability. Patients with relatively strong motor performance may be perceived as recovering well, and discharge planning may therefore place less emphasis on persistent cognitive-behavioral needs or reduce referral to post-acute services [4,14]. One possible explanation is that physical capability may outpace judgment and self-monitoring. Reduced insight and executive dysfunction, although not directly measured in the TBIMS dataset, may contribute to safety problems, ineffective problem-solving, and inconsistent engagement with services [10,11,14]. This pattern may help explain the lower odds of favorable disability status at 2 years despite better motor scores. The findings support discharge planning strategies that explicitly document cognitive risk and promote appropriate follow-up, including caregiver education and neuropsychological assessment, rather than relying on motor recovery as a proxy for global recovery. Because TBI is increasingly recognized as a chronic condition requiring longitudinal management [3,4], these results support a more domain-targeted approach to post-acute planning. In practical terms, a motor-dominant patient’s relatively strong physical performance at discharge may mask underlying cognitive vulnerability, potentially reducing clinical vigilance and referral to cognitive or neuropsychological services. Proactively documenting cognitive risk and establishing structured follow-up at discharge may therefore be particularly important for this group. In contrast, patients with a cognitive-dominant profile may benefit from interventions that leverage preserved cognitive capacity to support long-term adaptation and self-management.

In the stratified analyses, between-group differences were more evident in moderate discharge severity strata. In this study, moderate discharge severity referred to the prespecified intermediate strata used in sensitivity analyses, including discharge FIM total scores of 60–90 and discharge DRS scores of 4–6. This finding may indicate greater opportunity for divergence in recovery trajectories at intermediate severity, whereas severe baseline disability and ceiling effects in milder cases may reduce measurable differences between imbalance patterns. Accordingly, cognitive–motor imbalance phenotypes may be better interpreted as contextual indicators for monitoring and planning rather than deterministic prognostic labels.

No significant differences were identified in GOSE or PART-O outcomes. These measures are strongly influenced by contextual factors such as family support, environmental barriers, vocational opportunity, and access to community-based services, which may not be fully captured in registry data [16,20]. In addition, GOSE and PART-O may be less sensitive to subtle cognitive-behavioral differences in higher-functioning individuals. Broad response categories and potential ceiling effects may reduce between-group separation even when impairment- and activity-level differences are detectable [8,20]. Participation may also continue to evolve beyond 2 years, and longer follow-up with more sensitive psychosocial and cognitive-behavioral measures may reveal additional divergence.

Several limitations should be considered. First, outcome analyses used a complete-case approach at each follow-up time point, and the analytic sample therefore varied across outcomes and time points. Exclusion of participants with missing follow-up data may have introduced selection bias if missingness was related to prognosis, access to care, or psychosocial factors [16], and may have affected the comparability of matched groups over time. Second, although the TBIMS employs standardized data collection protocols, the multicenter nature of the registry may introduce inter-site and between-rater variability in outcome assessment, which could affect measurement consistency across centers. Third, cognitive–motor imbalance was operationalized using FIM subscale scores and an a priori threshold selected on pragmatic grounds, which was not derived from or validated against external clinical outcome data. These groups should therefore be interpreted as operational phenotypes rather than validated clinical categories. Similarly, the discharge FIM total strata used in sensitivity analyses were pragmatic and intended to generate clinically interpretable subgroups with sufficient sample size; alternative cutoffs may have produced different subgroup findings. Fourth, as an observational study, residual confounding from unmeasured factors cannot be excluded despite propensity score matching and covariate adjustment. Such factors may include neuroanatomical injury characteristics, premorbid cognitive reserve [27,28], psychiatric or behavioral comorbidity, and rehabilitation dose or content during and after inpatient rehabilitation. Finally, because the analytic cohort was created by recombining two pairwise matched sets, residual differences between the cognitive-dominant and motor-dominant groups should be considered when interpreting exploratory direct comparisons. Future studies incorporating neuroimaging, more sensitive cognitive-behavioral measures, and longer follow-up are needed to clarify the mechanisms underlying imbalance profiles and to refine phenotype definitions.

## 5. Conclusions

Discharge cognitive–motor imbalance patterns were associated with modest but potentially actionable differences in disability severity and functional independence over 2 years after moderate-to-severe TBI. Although overall discharge severity remained the main prognostic driver, the direction of imbalance provided additional domain-relevant information that may help identify patients who could benefit from targeted discharge planning and structured follow-up, particularly motor-dominant individuals with less readily recognized cognitive vulnerability despite relatively strong motor performance. Incorporating cognitive–motor imbalance phenotypes into severity-based discharge planning may improve risk stratification and support more tailored post-acute surveillance and service referral.

## Figures and Tables

**Table 1:** Baseline characteristics of patients before and after propensity score matching.

	Before Matching					After Matching				
Variable	Balanced (*n* = 974)	Cognitive (*n* = 2484)	Motor (*n* = 4884)	*p*	SMD	Balanced (*n* = 262)	Cognitive (*n* = 524)	Motor (*n* = 524)	*p*	SMD
Age, years	40.0 ± 19.7	40.9 ± 19.7	38.2 ± 17.1	<0.001	0.096	42.9 ± 19.5	42.6 ± 19.2	43.1 ± 18.2	0.91	0.017
Sex, male	670 (68.8)	1660 (66.8)	3749 (76.8)	<0.001	0.148	169 (64.5)	342 (65.3)	347 (66.2)	0.88	0.024
Race				0.22	0.073				0.83	0.050
White	500 (76.8)	1235 (74.3)	2572 (74.2)			184 (70.2)	367 (70.0)	359 (68.5)		
Black	93 (14.3)	300 (18.1)	616 (17.8)			47 (17.9)	92 (17.6)	106 (20.2)		
Others	58 (8.9)	127 (7.6)	276 (8.0)			31 (11.8)	65 (12.4)	59 (11.3)		
Marital status				0.046	0.055				0.97	0.031
Single	458 (47.1)	1119 (45.1)	2370 (48.6)			114 (43.5)	223 (42.6)	235 (44.8)		
Married	315 (32.4)	869 (35.0)	1556 (31.9)			94 (35.9)	191 (36.5)	184 (35.1)		
Previously married	200 (20.6)	493 (19.9)	951 (19.5)			54 (20.6)	110 (21.0)	105 (20.0)		
Education				0.002	0.081				0.69	0.063
Low	100 (12.0)	227 (10.4)	517 (12.9)			29 (11.1)	54 (10.3)	68 (13.0)		
Middle	393 (47.2)	1068 (48.9)	2033 (50.6)			109 (41.6)	228 (43.5)	213 (40.6)		
High	340 (40.8)	888 (40.7)	1467 (36.5)			124 (47.3)	242 (46.2)	243 (46.4)		
Employed	481 (60.6)	1251 (60.3)	2461 (65.0)	0.001	0.065	163 (62.2)	327 (62.4)	318 (60.7)	0.83	0.024
Mechanism				<0.001	0.106				0.49	0.048
Motor vehicle accident	511 (52.6)	1500 (60.4)	2582 (53.0)			121 (46.2)	256 (48.9)	237 (45.2)		
Fall	258 (26.5)	530 (21.4)	1145 (23.5)			75 (28.6)	145 (27.7)	150 (28.6)		
Assault	96 (9.9)	194 (7.8)	665 (13.6)			30 (11.5)	50 (9.5)	59 (11.3)		
GCS total	11.3 ± 4.2	11.4 ± 4.2	10.7 ± 4.0	<0.001	0.112	11.0 ± 4.3	11.0 ± 4.3	11.2 ± 3.9	0.71	0.034
Alcohol use	527 (61.4)	1279 (56.7)	2730 (64.8)	<0.001	0.111	168 (64.1)	340 (64.9)	335 (63.9)	0.95	0.013
Discharge FIM total	86.0 ± 31.4	83.1 ± 23.4	97.1 ± 18.3	<0.001	0.400	91.8 ± 27.1	90.6 ± 19.5	93.0 ± 19.7	0.19	0.074

Values are presented as mean ± standard deviation for continuous variables and number (%) for categorical variables. Abbreviations: SMD, standardized mean difference; GCS, Glasgow Coma Scale; FIM, Functional Independence Measure.

**Table 2:** Primary and secondary outcomes for functional recovery.

		Cognitive-Dominant		Motor-Dominant	
Category	Outcome	β or OR (95% CI)	*p*-Value	β or OR (95% CI)	*p*-Value
DRS	DRS, 1 year	−0.45 (−0.81, −0.09)	0.02	−0.10 (−0.46, 0.26)	0.60
	DRS, 2 years	−0.40 (−0.78, −0.03)	0.03	0.07 (−0.31, 0.44)	0.72
	DRS ≤ 3, 1 year	1.17 (0.78, 1.75)	0.45	0.84 (0.56, 1.26)	0.40
	DRS ≤ 3, 2 years	0.69 (0.40, 1.17)	0.17	0.52 (0.31, 0.88)	0.01
	DRS ≤ 3 at both 1 and 2 years	1.04 (0.74, 1.46)	0.82	0.80 (0.57, 1.13)	0.21
FIM	FIM total, 1 year	3.05 (1.11, 4.99)	0.002	2.81 (0.87, 4.76)	0.005
	FIM total, 2 years	2.05 (0.06, 4.04)	0.04	1.62 (−0.36, 3.61)	0.11
	FIM cognitive, 1 year	1.07 (0.46, 1.69)	<0.001	−0.61 (−1.23, 0.01)	0.05
	FIM cognitive, 2 years	0.92 (0.32, 1.52)	0.002	−0.59 (−1.19, 0.01)	0.05
	FIM motor, 1 year	2.00 (0.48, 3.52)	0.010	3.37 (1.85, 4.89)	<0.001
	FIM motor, 2 years	1.14 (−0.43, 2.71)	0.16	2.22 (0.85, 3.59)	0.002

Reference group:balanced group. Values represent β coefficients from linear regression models for continuous outcomes and odds ratios (ORs) from logistic regression models for binary outcomes (DRS ≤ 3). Models were adjusted for age, sex, race, marital status, education, employment status, injury mechanism, Glasgow Coma Scale (GCS), alcohol history, and discharge Functional Independence Measure (FIM) total score. Abbreviations: CI, confidence interval; DRS, Disability Rating Scale; FIM, Functional Independence Measure; OR, odds ratio.

**Table 3:** Exploratory outcomes for global function and societal participation.

		Cognitive-Dominant		Motor-Dominant	
Category	Outcome	β (95% CI)	*p*-Value	β (95% CI)	*p*-Value
GOSE	GOSE, 1 year	0.01 (−0.22, 0.24)	0.93	−0.11 (−0.34, 0.12)	0.34
	GOSE, 2 years	−0.02 (−0.25, 0.21)	0.87	−0.17 (−0.40, 0.06)	0.15
PART-O	Out & About, 1 year	0.08 (−0.05, 0.22)	0.24	0.05 (−0.08, 0.19)	0.44
	Out & About, 2 years	0.03 (−0.11, 0.16)	0.72	−0.05 (−0.18, 0.09)	0.47
	Productivity, 1 year	−0.07 (−0.22, 0.09)	0.41	−0.06 (−0.21, 0.10)	0.48
	Productivity, 2 years	−0.08 (−0.22, 0.07)	0.31	−0.06 (−0.21, 0.08)	0.39
	Social, 1 year	0.13 (−0.02, 0.29)	0.08	0.06 (−0.09, 0.22)	0.41
	Social, 2 years	0.08 (−0.07, 0.22)	0.31	0.02 (−0.12, 0.16)	0.77

Reference group:balanced group. Values represent β coefficients from linear regression models. Models were adjusted for age, sex, race, marital status, education, employment status, injury mechanism, Glasgow Coma Scale (GCS), alcohol history, and discharge Functional Independence Measure (FIM) total score. Abbreviations: CI, confidence interval; GOSE, Glasgow Outcome Scale—Extended; PART-O, Participation Assessment with Recombined Tools—Objective.

## Data Availability

The data used in this study are available from the Traumatic Brain Injury Model Systems (TBIMS) National Database. Access to the data requires application and approval from the TBIMS National Data and Statistical Center.

## Supplementary Materials

The following supporting information can be downloaded at: https://www.mdpi.com/article/10.3390/jcm15093249/s1, Figure S1: Propensity score distributions before and after propensity score matching; Table S1: Stratified analyses of outcomes by discharge severity subgroups.

## Abbreviations

The following abbreviations are used in this manuscript:

TBI	Traumatic Brain Injury
FIM	Functional Independence Measure
DRS	Disability Rating Scale
GOSE	Glasgow Outcome Scale—Extended
PART-O	Participation Assessment with Recombined Tools—Objective
TBIMS	Traumatic Brain Injury Model Systems
