# Supplementary material for: Discharge Cognitive–Motor Imbalance Patterns and Long-Term Outcomes After Traumatic Brain Injury: A Propensity Score-Matched Cohort Study
Source: J Clin Med. 2026 Apr 24;15(9):3249. doi: 10.3390/jcm15093249 (PMC13164557; doi:10.3390/jcm15093249)
Supplement: Supplementary file 1 [file jcm-15-03249-s001.zip › jcm-4249083-supplementary.pdf]

Supplementary Material

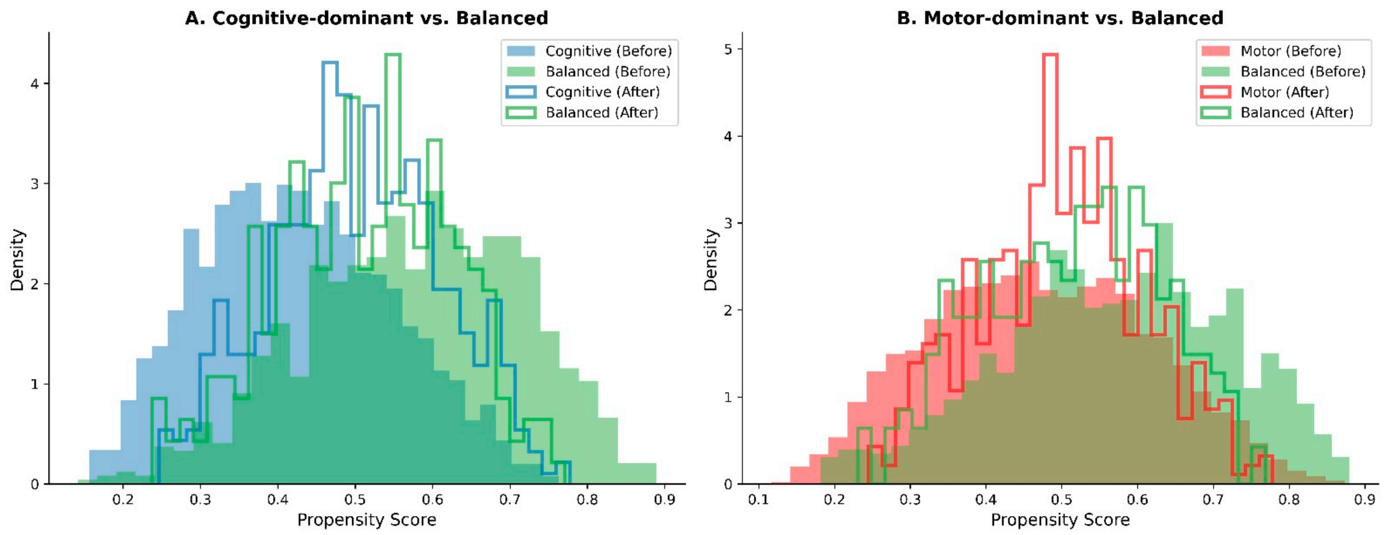

Figure S1. Propensity score distributions before and after propensity score matching

Panel A compares the cognitive-dominant group with the balanced group, and panel B compares the motor-dominant group with the balanced group. Filled histograms represent distributions before matching, whereas outlined histograms represent distributions after matching. Propensity score matching (PSM) was performed using a 1:2 matching ratio.

Table S1. Stratified analyses of outcomes by discharge severity subgroups.

		Cognitive-dominant		Motor-dominant	
**Outcome**	**Stratum**	**β (95% CI)**	**P value**	**β (95% CI)**	**P value**
DRS, 1 year	**FIM at discharge**				
	**Severe**	-1.24 (-3.84, 1.35)	0.35	-1.39 (-4.22, 1.44)	0.34
	**Moderate**	0.25 (-0.47, 0.97)	0.49	0.81 (0.09, 1.53)	0.03
	**Mild**	0.01 (-0.28, 0.30)	0.94	0.15 (-0.16, 0.47)	0.35
	**DRS at discharge**				
	**Severe**	-0.55 (-1.60, 0.49)	0.30	-0.10 (-1.10, 0.90)	0.85
	**Moderate**	0.20 (-0.18, 0.58)	0.31	0.22 (-0.16, 0.60)	0.26
	**Mild**	0.00 (-0.52, 0.53)	0.99	0.10 (-0.44, 0.64)	0.72
DRS, 2 years	**FIM at discharge**				
	**Severe**	-1.18 (-3.85, 1.49)	0.39	0.23 (-2.81, 3.26)	0.88
	**Moderate**	-0.07 (-0.92, 0.78)	0.86	0.42 (-0.44, 1.28)	0.33
	**Mild**	0.09 (-0.24, 0.42)	0.57	0.35 (0.03, 0.67)	0.03
	**DRS at discharge**				
	**Severe**	-1.16 (-2.27, -0.05)	0.04	-0.15 (-1.18, 0.88)	0.77
	**Moderate**	0.36 (-0.05, 0.77)	0.08	0.53 (0.12, 0.94)	0.01
	**Mild**	-0.26 (-0.82, 0.30)	0.36	-0.31 (-0.88, 0.25)	0.28
DRS ≤3, 1 year	**FIM at discharge**				
	**Severe**	5.53 (0.49, 62.97)	0.17	8.93 (0.71, 111.74)	0.09
	**Moderate**	0.79 (0.47, 1.31)	0.36	0.53 (0.32, 0.89)	0.02
	**Mild**	1.31 (0.80, 2.15)	0.29	1.19 (0.74, 1.90)	0.48
	**DRS at discharge**				
	**Severe**	1.45 (0.68, 3.08)	0.34	1.00 (0.50, 2.03)	0.99
	**Moderate**	0.84 (0.48, 1.47)	0.54	0.77 (0.43, 1.36)	0.36
	**Mild**	1.23 (0.29, 5.32)	0.78	1.01 (0.21, 4.90)	0.99
DRS ≤3, 2 years	**FIM at discharge**				
	**Severe**	0.87 (0.09, 8.17)	0.91	0.19 (0.01, 2.69)	0.22

	**Moderate**	0.79 (0.36, 1.73)	0.56	0.59 (0.27, 1.26)	0.17
	**Mild**	0.46 (0.18, 1.14)	0.09	0.46 (0.19, 1.13)	0.09
	**DRS at discharge**				
	**Severe**	1.60 (0.62, 4.09)	0.33	0.72 (0.31, 1.72)	0.46
	**Moderate**	0.27 (0.11, 0.66)	0.004	0.30 (0.12, 0.73)	0.008
	**Mild**	2.45 (0.42, 14.36)	0.32	6.31 (0.68, 58.31)	0.11
**FIM total, 1 year**	**FIM at discharge**				
	**Severe**	4.21 (-5.89, 14.31)	0.42	-4.52 (-15.58, 6.54)	0.42
	**Moderate**	2.38 (0.14, 4.62)	0.04	2.52 (0.27, 4.77)	0.03
	**Mild**	1.68 (-0.33, 3.69)	0.10	2.35 (0.28, 4.42)	0.03
	**DRS at discharge**				
	**Severe**	2.68 (-0.78, 6.14)	0.13	2.56 (-0.84, 5.96)	0.14
	**Moderate**	2.60 (0.21, 4.99)	0.03	2.64 (0.25, 5.03)	0.03
	**Mild**	2.53 (-0.97, 6.03)	0.16	4.06 (0.45, 7.67)	0.03
**FIM cognitive, 1 year**	**FIM at discharge**				
	**Severe**	1.09 (-3.36, 5.53)	0.63	-1.59 (-6.42, 3.25)	0.52
	**Moderate**	1.15 (0.33, 1.96)	0.006	-0.65 (-1.47, 0.17)	0.12
	**Mild**	0.70 (-0.13, 1.53)	0.10	-0.41 (-1.29, 0.48)	0.37
	**DRS at discharge**				
	**Severe**	2.28 (1.10, 3.45)	<0.001	-0.45 (-1.60, 0.70)	0.44
	**Moderate**	0.63 (-0.15, 1.41)	0.11	-0.40 (-1.18, 0.38)	0.32
	**Mild**	0.31 (-0.99, 1.62)	0.64	-0.82 (-2.17, 0.53)	0.23
**FIM motor, 1 year**	**FIM at discharge**				
	**Severe**	3.31 (-6.19, 12.81)	0.50	-5.13 (-15.45, 5.19)	0.33
	**Moderate**	1.24 (-0.39, 2.88)	0.14	3.16 (1.53, 4.80)	<0.001
	**Mild**	0.98 (-0.22, 2.17)	0.11	2.21 (0.97, 3.44)	<0.001
	**DRS at discharge**				
	**Severe**	0.42 (-1.89, 2.74)	0.72	2.65 (0.38, 4.93)	0.02
	**Moderate**	1.97 (0.37, 3.57)	0.02	3.04 (1.44, 4.64)	<0.001
	**Mild**	2.26 (-0.42, 4.94)	0.10	4.23 (1.45, 7.02)	0.003

Reference group: balanced group. Values represent β coefficients from linear regression models and odds ratios (ORs) from logistic regression models. Severity strata were defined as follows: discharge Functional Independence Measure (FIM) (severe, <60; moderate, 60–90; mild, >90) and discharge Disability Rating Scale (DRS) (severe, ≥7; moderate, 4–6; mild, ≤3). Sample sizes for discharge FIM strata were: severe (n=103), moderate (n=418), and mild (n=769). Sample sizes for discharge DRS strata were: severe (n=358), moderate (n=706), and mild (n=224). Higher β indicates better outcomes for FIM outcomes, whereas lower β indicates lower disability for continuous DRS outcomes. For binary outcomes (DRS ≤3), OR >1 indicates higher odds of a favorable outcome. Models were adjusted for age, sex, race, marital status, education, employment status, injury mechanism, Glasgow Coma Scale (GCS), alcohol history, and discharge FIM total score. Subgroup analyses were exploratory, and no adjustment for multiple comparisons was applied. Abbreviations: β, regression coefficient; OR, odds ratio; CI, confidence interval; DRS, Disability Rating Scale; FIM, Functional Independence Measure; GCS, Glasgow Coma Scale.
